# Complementary genetic and genomic approaches help characterize the linkage group I seed protein QTL in soybean

**DOI:** 10.1186/1471-2229-10-41

**Published:** 2010-03-03

**Authors:** Yung-Tsi Bolon, Bindu Joseph, Steven B Cannon, Michelle A Graham, Brian W Diers, Andrew D Farmer, Gregory D May, Gary J Muehlbauer, James E Specht, Zheng Jin Tu, Nathan Weeks, Wayne W Xu, Randy C Shoemaker, Carroll P Vance

**Affiliations:** 1 United States Department of Agriculture-Agricultural Research Service, Plant Research Unit, St Paul, MN 55108, USA; 2 Department of Agronomy, Iowa State University, Ames, IA 50011, USA; 3 United States Department of Agriculture-Agricultural Research Service, Corn Insects and Crop Genetics Research Unit, Ames, IA 50011, USA; 4 Department of Crop Sciences, University of Illinois, 1101 West Peabody Dr, Urbana, IL 61801, USA; 5 National Center for Genome Resources, Santa Fe, NM 87505, USA; 6 Department of Agronomy and Plant Genetics, University of Minnesota, St Paul, MN 55108, USA; 7 Department of Agronomy, University of Nebraska, Lincoln, NE 68583, USA; 8 Minnesota Supercomputing Institute, University of Minnesota, Minneapolis, MN 55455, USA

## Abstract

**Background:**

The nutritional and economic value of many crops is effectively a function of seed protein and oil content. Insight into the genetic and molecular control mechanisms involved in the deposition of these constituents in the developing seed is needed to guide crop improvement. A quantitative trait locus (QTL) on Linkage Group I (LG I) of soybean (*Glycine max *(L.) Merrill) has a striking effect on seed protein content.

**Results:**

A soybean near-isogenic line (NIL) pair contrasting in seed protein and differing in an introgressed genomic segment containing the LG I protein QTL was used as a resource to demarcate the QTL region and to study variation in transcript abundance in developing seed. The LG I QTL region was delineated to less than 8.4 Mbp of genomic sequence on chromosome 20. Using Affymetrix^® ^Soy GeneChip and high-throughput Illumina^® ^whole transcriptome sequencing platforms, 13 genes displaying significant seed transcript accumulation differences between NILs were identified that mapped to the 8.4 Mbp LG I protein QTL region.

**Conclusions:**

This study identifies gene candidates at the LG I protein QTL for potential involvement in the regulation of protein content in the soybean seed. The results demonstrate the power of complementary approaches to characterize contrasting NILs and provide genome-wide transcriptome insight towards understanding seed biology and the soybean genome.

## Background

Seed protein and oil are crucial to the value of many crop species. During seed development, carbon and nitrogen are partitioned among protein, oil, and carbohydrates [1-6]. In legumes, particularly soybean (*Glycine max *(L.) Merrill), protein and oil are primary nutritional components of mature seed. Protein and oil comprise some 40% and 20%, respectively, of soybean seed. Protein meal is a major byproduct of soybean processing, and high seed protein content allows processors to derive meal with high nutritional value [7]. A better understanding of the genetic basis of seed protein variation is important for developing strategies to improve seed quality traits not only in soybean but also in other legumes and cereal grains.

Storage reserves account for the majority of the protein in the seed [8,9]. The period of seed development where these reserves accumulate is commonly referred to as the seed filling stage, a 4- to 5-week period of cell expansion that occurs once cell division is complete [10]. The most prevalent seed storage proteins in soybean are beta-conglycinin and glycinin [11,12]. A number of diverse and interlinked processes, including photosynthesis, sucrose signaling, and transport, are associated with seed development and the regulation of complex traits [2,13,14].

Genetic control of seed constituents and size is inherited in a quantitative manner. Many quantitative trait loci (QTLs) associated with seed protein and size have been identified in several species including wheat [15], Arabidopsis [16], rice [17], pea [18], and barley [5]. In soybean, numerous QTLs associated with protein have been identified [19-23]. The seed protein QTL mapped to soybean linkage group I (LG I) is of particular interest due to the large additive effect that accounts for its consistent detection in many soybean mapping populations [22,24,25] and across multiple environments [26]. Inheritance of the high protein allele from *G. soja *at LG I resulted in a seed protein increase of 18 to 24 g/kg, and this increase was also associated with lower oil concentration [24,25,27]; a negative phenotypic correlation between soybean seed protein and oil content is well documented [28-31]. Nichols et al. [32] fine mapped the LG I protein QTL region to a 3 cM interval using BC_5_F_5_-derived near-isogenic lines (NILs) contrasting in seed protein and oil. Although linkage analysis is a valuable tool for localizing genetic regions of interest for a trait, the capabilities of mapping can be greatly enhanced by genomic approaches to identify genes that may control these traits.

Analyses of transcript profiles by microarrays have provided insight into the genes and processes involved in developing seed of Arabidopsis [33,34], soybean [35-37], *Medicago truncatula *[4,38,39], wheat [40], barley [5,41], and rice panicles [42]. Transcript changes, especially when used to contrast NILs, have proven useful for the discovery of genes of interest in soybean and other species [5,43-45].

In the present study, we leveraged a combination of resources - a NIL pair that differed substantially in seed protein [32], transcript profiling by Affymetrix^® ^Soy GeneChip microarray, Illumina^® ^high-throughput transcriptome sequencing platforms, and the newly available soybean genome sequence--to assess genomic and genetic contributions to seed protein traits in soybean. The objectives of our study were to: 1) define the borders of the genomic segment encompassing the LG I protein QTL region, 2) characterize transcript accumulation in the developing seed of a NIL pair known to produce contrasting final seed protein content, and 3) identify candidate genes for this seed protein QTL. The accomplishment of these objectives constitutes the first step toward understanding the genetic and molecular mechanisms underlying the regulation of seed protein. In addition, the large dataset provided through this study is a valuable tool for further analysis of the soybean transcriptome.

## Results

### Demarcation of the QTL region

**Figure 1:**
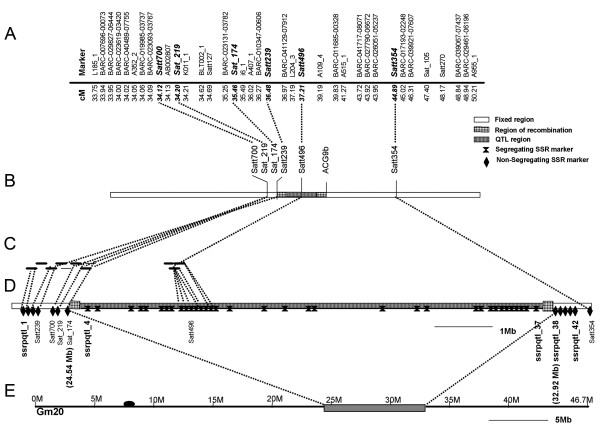
**Demarcation of the LG I QTL region**. (A) The genetic map of LG I [47] shows the markers that mapped close to the QTL region. (B) The fine map of the QTL [32] shows the QTL position in the segregating region between SSR marker Satt239 and AFLP marker ACG9b. (C) The physical map of the QTL region shows where the BACs were anchored to the SSR markers (Satt239, Satt700, Sat_174, Sat_219, and Satt496). BACs shown as bold lines were sequenced. BACs shown as thin lines were not sequenced; only BAC end sequences were generated. (D) Demarcation of the QTL region on chromosome 20 (Gm20) using additional SSR markers. The new SSR markers were named ssrpqtl_1 through ssrpqtl_42 (in bold) according to ascending position on chromosome 20 (see also Additional file 1: Table S1). The position of the LG I protein QTL region is demarcated between 24.54 Mb (Sat_174) and 32.92 Mb (ssrpqtl_38). (E) The QTL region highlighted on Chromosome 20. The dark oval represents the position of the centromere.

Previous genetic studies [27,32] localized the LG I protein QTL region to a 3 cM interval. NIL populations used to map the LG I protein QTL were created by backcrossing the high protein *G. soja *(PI468916) allele into a *G. max *background (A81-356022) [32]. The NIL population P-C609-45-2 was found to segregate for the smallest LG I QTL interval corresponding to high and low seed protein phenotypes in the field [32]. In this study, these NILs were used to link the genetic map (Figures 1A and 1B) to the physical map (Figures 1C and 1E) and to identify recombination break points in P-C609-45-2 to demarcate the protein QTL region (Figure 1D).

To obtain a physical map of the protein QTL region, BAC (bacterial artificial chromosome) libraries of soybean genomic DNA were scanned for alignment to known markers, and a BAC-based physical map was assembled to span markers Satt239 and Satt496 (Figure 1). This BAC-based map accounted for approximately 1.2 Mb of the QTL region. Newly derived SSR (simple sequence repeat) markers from the BAC sequence that were polymorphic between A81-356022 and PI468916 were screened to determine if they segregated in the P-C609-45-2 population. Because the introgressed QTL-containing segment was segregating in the P-C609-45-2 population, markers located in that region were expected to segregate in the population. Upon release of the soybean whole genome sequence, alignment of BAC sequences to the soybean whole genome assembly (version Glyma1, [46]) identified chromosome 20 as the best match to all the BACs in the LG I protein QTL physical map. The order of BAC sequence alignment to chromosome 20 was in agreement with the physical map (Figures 1C, D, and 1E).

Forty-eight SSR markers (Figure 1D), including 42 SSR markers (see Additional file 1) derived from the BAC sequences and from the whole genome sequence spanning the QTL region plus six previously genetically mapped SSR markers [47], were screened for segregation as described above. Thirty-four of the 42 SSR markers derived in this study segregated in the P-C609-45-2 population. The high and low protein phenotypes of the segregating progeny corresponded to the expected parental marker alleles originating from the high and low protein parents [32].

The protein QTL region was delineated to approximately 8.4 Mbp of genomic sequence between Sat_174 and ssrpqtl_38, the two closest non-segregating SSR markers flanking the left and right borders of the protein QTL region on chromosome 20 (Figure 1D). The coordinates of the borders stretch from 24.54 Mb to 32.92 Mb on chromosome 20.

### Phenotypic evaluation of seed protein and oil in NILs

**Figure 2:**
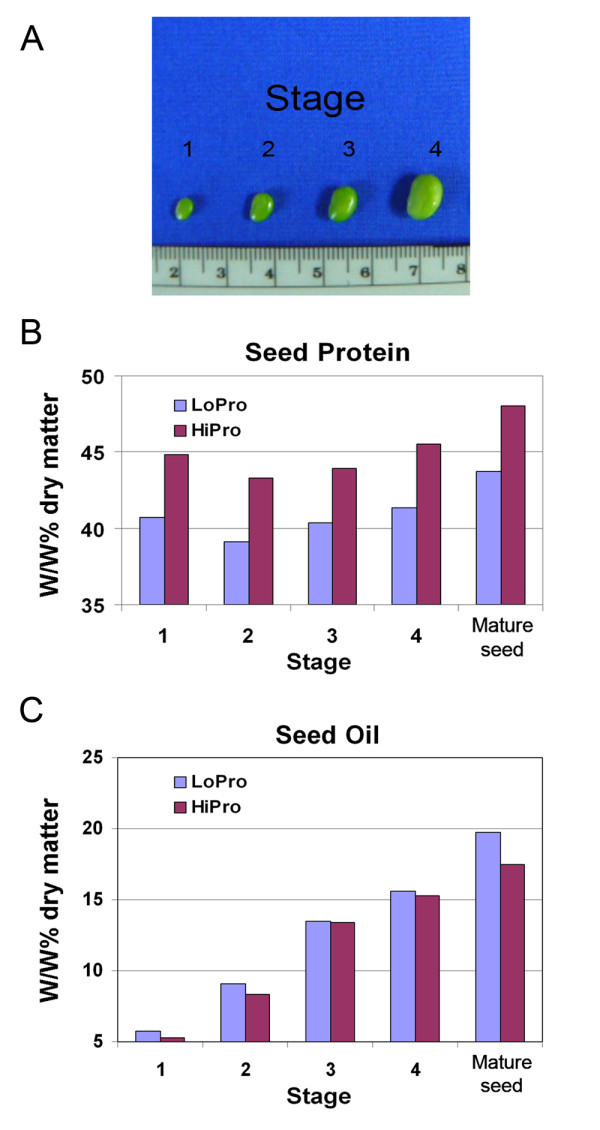
**Phenotypic evaluation of NILs**. (A) Different stages of the developing soybean seed are shown. Stages one to four correspond to the seed fill stages that were harvested for phenotypic evaluation and concurrently used for gene expression profiling in this study. Stage 1 = 25 to 50 mg seed. Stage 2 = >50 to 100 mg seed. Stage 3 = >100 to 200 mg seed. Stage 4 = >200 to 300 mg seed. Shown in the diagram are 25 mg, 50 mg, 100 mg, and 200 mg seed sizes. (B) Crude protein profiles graphed on a w/w% dry matter basis for the different stages of developing seed (stages one to four) and the final mature soybean seed. Protein profiles are graphed for both the low protein line (LoPro) and the high protein line (HiPro). (C) Crude oil profiles graphed on a w/w% dry matter basis for the different stages of developing seed (stages one to four) and the final mature soybean seed.

A NIL pair derived from the P-C609-45-2 population was chosen for further study. One line (LoPro = LD0-15146) retained the *G. max *(A81-356022) background at the LG I protein QTL region, and the other (HiPro = LD0-15154) inherited the high protein allele in that region from *G. soja *(PI468916). The protein and oil phenotypes in the NIL pair were evaluated at four stages of seed fill (Figure 2A). These four stages during seed fill were defined by seed size and were harvested at the same time during the R5 stage of development from the same plants for direct comparison. Stage one corresponded to a seed size of 25 to 50 mg, stage two to greater than 50 to 100 mg seed, stage three to greater than 100 to 200 mg seed, and stage four to greater than 200 to 300 mg seed. At stage one, seed organs and tissues are formed but have yet to increase in cell size (data not shown). It is noteworthy that seed protein differences between LoPro (low protein line, homozygous for A81-356022) and HiPro (high protein line, homozygous for PI468916) genotypes were apparent at the earliest stage of evaluation (Figure 2B). Moreover, that difference remained consistent through the subsequent stages. Seed oil values, however, did not show as marked a contrast in the early stages (Figure 2C). The protein and oil phenotypes for the NILs at seed maturity were consistent with the previously reported values (Figures 2B, C; stage 5).

### Transcript accumulation changes during seed fill

To examine transcript accumulation changes during seed fill, transcript profiles were evaluated in seeds of each genotype (LoPro or HiPro) from the four stages above by Soy Genome Affymetrix^® ^GeneChip analyses. Out of 37,701 soybean probesets on the GeneChip, 64-69% were defined as 'present' in three out of three replicates by MAS5 analysis of the various seed stages in both genotypes. These detection figures are comparable to those found in seed microarray studies of other species [34,38]. Differences in the transcriptomes of the NIL pair may reflect or affect the high and low protein and oil phenotypes seen in the lines. Using Student's *t*-test to evaluate significance, Affymetrix^® ^GeneChip probesets with at least 1.5-fold change between stages were identified at an FDR (false discovery rate) of less than 5% [48]. Transcript accumulation changes across stages were evaluated with reference to the stage one profiles (stage two vs. stage one, stage three vs. stage one, stage four vs. one). In both genotypes, no probesets from the stage two versus stage one comparison qualified under the FDR < 0.05 criterion, so this comparison was excluded from further analysis. The number of probesets representing differentially accumulated transcripts with higher accumulation in stage three compared to stage one was greater in HiPro than in LoPro (716 vs. 616), and this difference was again apparent between stages four and one (2094 vs. 1294) (see Additional files 2 and 3).

Analysis of all probeset expression changes revealed that 18.2% of the genes that significantly increased in expression over time in either genotype were shared between LoPro and HiPro (see Additional file 4). Transcripts common to both genotypes that increase significantly in stage four seed as opposed to stage one seed include: beta-conglycinins and glycinins, sucrose binding proteins, heat shock chaperonins, late embryogenesis messages, seed maturation proteins, glutathione S-transferases and peroxidases, iron binding and flavonoid synthesis proteins, and numerous transporters. Interestingly, 25 transcripts with ubiquitin-related annotations were found to increase in accumulation over time in both genotypes while three were found to decrease in accumulation levels in both genotypes (see Additional files 4 and 5). It is noteworthy that some 53 transcription factor messages showed enhanced abundance at stage three or four versus stage one seed.

Of the genes that decreased in expression over time, 30.2% of these genes were shared between LoPro and HiPro (see Additional file 5). Transcripts common to both genotypes that were reduced in abundance at stage four as opposed to stage one include genes involved in flavonoid metabolism, cell wall deposition, kinases (particularly those related to cell cycle), response to arachidonic acid, strictosidine synthesis, and disease resistance response. Twenty transcription factor annotations were common to both lines and displayed reduced abundance by stage three or four.

During seed development, the synthesis of seed storage products is coordinated with carbohydrate and nitrogen metabolic processes involving many transporters [49]. Some 26 transport-related transcripts increased in abundance in both genotypes, including gene transcripts annotated as ammonium, sugar, metal, and ion transporters (see Additional file 4). Meanwhile, some 33 transport-related transcripts decreased in accumulation levels in both genotypes, and these included transcripts annotated as ammonium, sugar, and ABC transporters (see Additional file 5).

A high number of microtubule-related gene transcripts were also found to decrease in abundance, supporting a role for fundamental transport mechanisms [50,51] and the slowing of cell expansion [52] during these stages of seed development. Eleven microtubule-related transcripts, including those involved in activity and movement, were found to decrease in abundance versus four microtubule-related transcripts that increased in abundance in both genotypes (see Additional files 4 and 5). Cyclin-related transcripts were also found in both genotypes. It is interesting to note that of the transcripts directly associated with cell division cycle annotations, those that increased in abundance included transcripts for Cdc48 and five transcripts annotated as tyrosine kinase specific for activated (GTP-bound) p21cdc42Hs (see Additional file 4). Those that decreased in abundance included transcripts for Cdc20 and Cdc50 (see Additional file 5). At least one transcript related to Cdc2 was found to accumulate in both directions for both genotypes over time (see Additional files 4 and 5).

Sucrose is well known for its many roles during seed development [3,53-55]. Sixteen transcripts with sucrose-related annotations were found to increase in accumulation in both genotypes, and these annotations included sucrose-binding protein and sucrose degradation and transport-related genes (see Additional file 4). This number is in contrast to the five sucrose-related transcripts that were found to decrease in accumulation in both genotypes and that included sucrose degradation and sucrose response genes (see Additional file 5).

Variation in transcriptome abundance profiles revealed differences between the two genotypes that may relate to their phenotypes. Tables 1 and 2 show the 15 transcripts that were most enhanced in abundance from each genotype in stage four seed versus stage one seed. Overall, HiPro possessed 200 transcripts with greater than four-fold abundance in stage four versus stage one seed, compared to 40 transcripts in LoPro. In addition, the HiPro line showed more than five times greater maximum fold change differences between stage four and stage one. HiPro revealed a striking abundance of transcripts related to protein accumulation, iron sequestration, sucrose binding, and seed maturation (Table 1). By comparison, the greatest abundance of transcripts in LoPro related to chaperonin heat shock protein, peptide transporter kinases, and glutathionine S-transferase (Table 2). Interestingly, transcripts related to chloroplast function were greatly reduced in LoPro in both abundance and unique representation in comparison to HiPro (see Additional files 6 and 7). Transcripts with accumulation changes were also assigned to gene ontology categories, and gene categories that were enriched under each condition within each genotype were identified (see Additional file 8).

**Table 1:** The 15 most highly upregulated Affymetrix^® ^probesets found in HiPro from stage one to stage four

Affy ID	*P *value	HiPro Stage 1	HiPro Stage 4	Ratio of Means Stage 4/Stage 1	Uniprot Description	E-value
Gma.1017.1.S1_at	1.57E-09	750	815093	1086.8	Cluster: Beta-conglycinin, beta chain precursor; *Glycine max*	0
Gma.1017.1.S1_s_at	4.06E-06	2032	952032	468.5	"	0
Gma.1017.2.S1_a_at	7.97E-06	5017	1160437	231.3	"	0
Gma.8531.1.S1_at	2.67E-05	3496	711843	203.6	Cluster: Seed maturation protein PM31; *Glycine max*	4 × 10^-87^
Gma.11119.2.S1_s_at	9.84E-05	1209	139593	115.4	Cluster: *G. max *mRNA from stress-induced gene; *Glycine max*	2 × 10^-79^
GmaAffx.48565.1.S1_at	8.72E-05	4270	421867	98.8	Cluster: Oxidoreductase, short chain dehydrogenase/reductase family, putative; *Medicago truncatula*	6 × 10^-56^
AFFX-Gm_SucBP_5_at	3.42E-07	3406	332671	97.7	Cluster: Sucrose-binding protein 2; *Glycine max*	0
Gma.10058.1.S1_at	5.23E-05	6724	510079	75.9	Cluster: Glycinin G3 precursor [Contains: Glycinin A subunit; Glycinin B subunit]; *Glycine max*	0
Gma.939.1.A1_at	1.31E-04	13550	833645	61.5	Cluster: Oxidoreductase, short chain dehydrogenase/reductase family, putative; *Medicago truncatula*	1 × 10^-65^
Gma.2505.1.S1_a_at	3.11E-05	2463	112324	45.6	Cluster: Ferritin-2, Chloroplast precursor; *Glycine max*	1 × 10^-142^
Gma.10.1.S1_at	5.93E-04	3516	141501	40.2	Cluster: Late embryogenesis-abundant protein; *Glycine max*	7 × 10^-54^
Gma.2505.1.S1_at	7.47E-05	5640	215422	38.2	Cluster: Ferritin-2, chloroplast precursor; *Glycine max*	1 × 10^-142^
GmaAffx.8078.1.S1_at	1.38E-04	7347	277493	37.8	Cluster: Expressed protein; *Oryza sativa *(japonica cultivar-group)	1 × 10^-19^
GmaAffx.24413.1.A1_at	1.01E-04	3559	117894	33.1	Rep: Pv42p - *Phaseolus vulgaris *(Kidney bean) (French bean)	1 × 10^-14^
Gma.8445.1.S1_at	5.03E-04	1066	34010	31.9	Cluster: Basic 7S globulin 2 precursor (Bg) (SBg7S) *Glycine max*	0

**Table 2:** The 15 most highly upregulated Affymetrix^® ^probesets found in LoPro from stage one to stage four

Affy ID	*P *value	LoPro Stage 1	LoPro Stage 4	Ratio of Means Stage 4/Stage 1	Uniprot Description	E-value
GmaAffx.35952.1.S1_at	6.07E-05	3847	573557	149.1	Cluster: Heat shock protein Hsp20; *Medicago truncatula*	2 × 10^-65^
Gma.4624.1.S1_s_at	2.83E-06	782	17143	21.9	Cluster: Specific tissue protein 1; *Cicer arietinum *(Chickpea)	1 × 10^-45^
GmaAffx.22552.1.S1_at	2.58E-04	2487	40880	16.4	Cluster: Putative peptide transporter; *Arabidopsis thaliana*	2 × 10^-55^
Gma.17917.1.S1_at	6.95E-05	707	7033	9.9	Cluster: Suspensor-specific protein; *Phaseolus coccineus*	5 × 10^-23^
Gma8516.1.S1_at	1.33E-05	3512	32139	9.2	Cluster: Glutathione S-transferase GST 11; *Glycine max*	1 × 10^-124^
soybean_rRNA_114_RC_at	4.66E-04	13887	121092	8.7		
GmaAffx.90956.1.S1_s_at	3.74E-05	22279	186953	8.4	Rep: At5 g54075 - *Arabidopsis thaliana*	5 × 10^-7^
GmaAffx.71277.1.S1_at	1.46E-04	2085	15568	7.5		
GmaAffx.39349.1.S1_at	2.26E-04	23625	166676	7.1	Cluster: Os12 g0514100 protein; *Oryza sativa *(japonica cultivar-group)	2 × 10^-17^
GmaAffx.34293.1.S1_at	3.92E-04	522	3482	6.7		
GmaAffx.87730.1.S1_at	9.35E-04	7132	45243	6.3	Cluster: Expressed protein; *Arabidopsis thaliana*	1 × 10^-12^
GmaAffx.75384.1.S1_at	2.26E-04	5443	33676	6.2		
Gma.8612.1.S1_at	7.46E-04	3282	19869	6.1	Cluster: predicted protein; *Magnaporthe grisea *70-15	4 × 10^-07^
Gma.12309.1.S1_at	6.90E-05	38247	228429	6.0	Cluster: Hypothetical protein F21F14.210; *Arabidopsis thaliana*	3 × 10^-99^
Gma.6617.1.S1_at	8.37E-05	2823	16373	5.8		

Transcripts for specific genes were also examined closely. The effect of Dof transcription factors on seed oil regulation have been previously documented [56], where GmDof4 and GmDof11 were found to contribute to high seed oil phenotypes in *Arabidopsis*. In our study, Dof22 and Dof24 genes were upregulated in the HiPro soy line, but no significant difference was seen in the transcript abundance for Dof4 and Dof11 in either genotype (data not shown).

### Differentially accumulated transcripts between NILs identified by microarray

**Figure 3:**
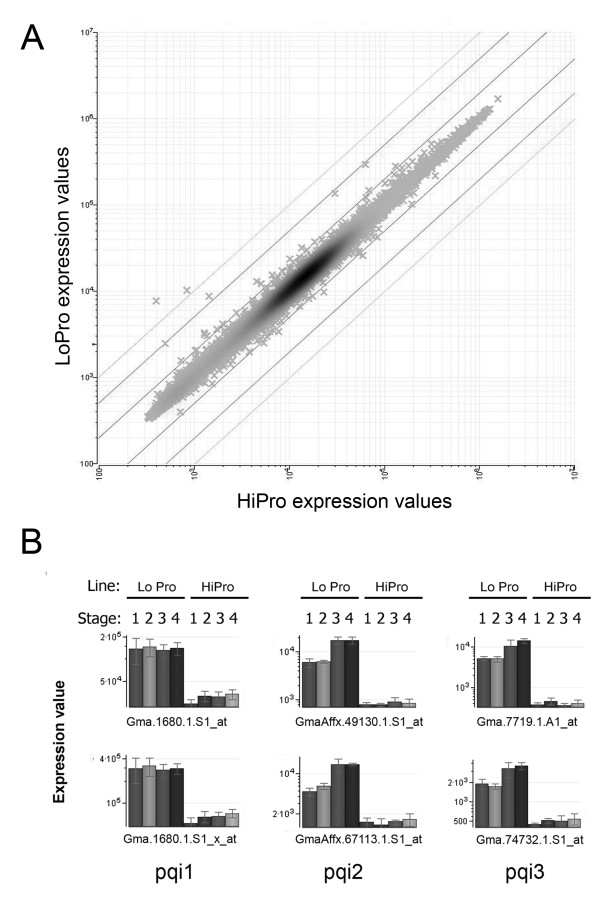
**Differentially accumulated transcripts between NILs detected by microarray**. (A) Log-log scatter plot of probeset expression values (x) from Student's *t*-test evaluation of combined stages from LoPro vs. HiPro highlighted six probesets with greater than four-fold change expression values. Diagonal lines represent two-fold, five-fold, and ten-fold change borders in either direction. (B) Expression values for the six probesets from (A) are graphed as a function of stage within each genotype. Standard error bars are shown for the three replicates. The six probesets correspond to a total of three genes (pqi1, pqi2, pqi3) represented by two Affymetrix^® ^probesets each. Probesets Gma.1680.1.S1_at and Gma.1680.1.S1_x_at represent pqi1, probesets GmaAffx.49130.1.S1_at and GmaAffx.67113.1.S1_at represent pqi2, and probesets Gma.7719.1.A1_at and Gma.74732.1.S1_at represent pqi3.

Direct comparisons of transcript accumulation between the two genotypes showed few significant differences by Soy Genome Affymetrix^® ^GeneChip analyses. Differentially expressed transcripts between the two genotypes were detected using Student's *t*-test. At a false discovery rate of 5% or less [48], only 13 Affymetrix^® ^probesets displayed at least 1.5-fold change between the two genotypes LoPro and HiPro (Table 3). Strikingly, six probesets were detected at greater than four-fold change between the two genotypes (Figure 3A). Examination of the six probesets above revealed that they likely represent three genes according to EST and GenBank data. These three genes are labeled as pqi1, pqi2, and pqi3 (Figure 3B). All six of the probesets with the greatest fold change were detected as transcripts with greater abundance in LoPro than in HiPro at all four stages (Figure 3A and 3B). Probesets representing transcripts with greater abundance in HiPro than in LoPro also existed (Table 3, Figure 3A).

**Table 3:** Differentially accumulated transcripts between LoPro and HiPro identified by Affymetrix^® ^Soy GeneChip

#	Affymetrix^® ^ID	LoPro	HiPro	Ratio of Means LoPro/HiPro	*P *value	FDR	Ch	Start	Stop	**Uniprot Desc**.	E-value
1	Gma.7719.1.A1_at	7705	401	19.22	2.43 × 10^-15^	9.16 × 10^-11^	20	26511887	26511422	Mov34/MPN/PAD-1	1 × 10^-19^
	GmaAffx.74372.1.S1_at	2478	502	4.94	2.63 × 10^-12^	1.98 × 10^-08^	20	26512404	26511978	"	"
2	Gma.1680.1.S1_x_at	289926	63810	4.54	5.51 × 10^-15^	1.04 × 10^-10^	20	32331958	32332062	Hypothetical protein	6 × 10^-24^
	Gma.1680.1.S1_at	134237	30039	4.47	1.05 × 10^-14^	1.32 × 10^-10^	20	32331999	32332062	"	"
3	GmaAffx.49130.1.S1_at	10163	842	12.06	4.38 × 10^-13^	4.12 × 10^-09^	20	30182754	30182479	na	na
	GmaAffx.67113.1.S1_at	8643	1438	6.01	2.19 × 10^-10^	9.17 × 10^-07^	20	30182353	30181939	"	"
4	GmaAffx.65278.1.A1_at	2695	5039	0.53	5.95 × 10^-12^	3.74 × 10^-08^	20	31053180	31054158	na	na
5	Gma.926.1.A1_at	2047	1031	1.99	2.99 × 10^-11^	1.61 × 10^-07^	20	31812657	31812894	na	na
6	GmaAffx.55722.1.S1_at	12024	5126	2.35	1.01 × 10^-10^	4.74 × 10^-07^	20	26515177	26514175	Hypothetical protein	2 × 10^-27^
7	GmaAffx.69807.1.A1_at	8369	13209	0.63	8.08 × 10^-07^	2.42 × 10^-03^	17	1020953	1020579	Hypothetical protein	3 × 10^-23^
8	Gma.10034.1.A1_at	1563	2548	0.61	8.35 × 10^-07^	2.42 × 10^-03^	18	13340405	13340201	na	na
9	GmaAffx.42487.1.S1_at	9513	5786	1.64	1.51 × 10^-06^	3.79 × 10^-03^	18	12183748	12183617	na	na
10	GmaAffx.47978.1.S1_at	3007	1378	2.18	2.31 × 10^-05^	4.58 × 10^-02^	7	897321	897126	Putative phosphatase	3 × 10^-75^

Each Affymetrix^® ^probeset identifier (ID) is shown with corresponding normalized expression values for LoPro and HiPro and the ratio of mean LoPro divided by mean HiPro values. Criteria for the list: FDR < 0.05 and fold-change > 1.5. The Uniprot description for each Affymetrix^® ^ID is accompanied by the E-value for the alignment; na = not applicable, no significant alignment.

An N-way ANOVA test was also conducted to examine transcript accumulation differences simultaneously across multiple factors, genotype, and time (stage) within the genotype. At FDR < 0.05 [48], a total of 66 Soy Affymetrix^® ^probesets were detected with differential changes in transcript accumulation using this method (see Additional file 9). Interestingly, five transcription factor-related transcripts, annotated as bZIP, ethylene-responsive, or heat shock, were detected with differential accumulation patterns (see Additional file 9). Again, the six probesets with the most highly differential accumulation values were represented (Table 3, Figure 3, see Additional file 9).

**Figure 4:**
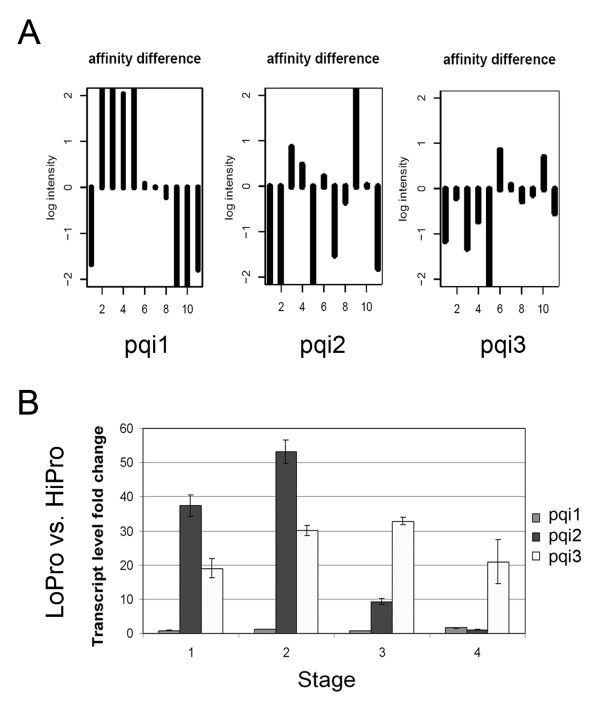
**Evaluation of differentially accumulated transcripts between NILs detected by microarray**. (A) Single feature polymorphism (SFP) evaluation of the probesets for the three genes selected from Figure 3. Plots show the log intensity of the affinity difference between LoPro and HiPro for each probe of the representative 11-member probeset for each gene. (B) Quantitative real-time reverse transcriptase-polymerase chain reaction (qRT-PCR) was performed in triplicate for each of the three genes. Transcript level fold changes were compared between LoPro and HiPro lines with reference to an actin control.

Because the Affymetrix^® ^GeneChip analysis was performed using transcripts from two different genotypes, the possibility of the presence of feature polymorphisms in the transcripts that could alter probe to transcript affinity was high. Therefore, single feature polymorphism (SFP) analysis [57] was performed using the Affymetrix^® ^GeneChip data and an algorithm based on the Li-Wong model [58] combined with a modified probe level statistical method [59]. SFP analysis of the three genes above showed large affinity differences to multiple probes on the Affymetrix^® ^GeneChip (Figure 4A). These three genes were potentially polymorphic in one or more regions between the two genotypes or completely absent in one genotype.

To further validate the microarray data, quantitative reverse transcriptase-polymerase chain reaction (qRT-PCR) was performed. Specific primers were designed for the three genes and an actin control. Significant differences between LoPro and HiPro were observed for pqi2 and pqi3 (Figure 4B). However, no significant transcript level fold changes were observed for pqi1 (Figure 4B). Thus, only two of the three genes identified as upregulated in LoPro in prior analyses were determined to display differentially accumulating transcripts between the two genotypes by qRT-PCR.

### Genes with differentially accumulated transcripts between NILs map to the LG I protein QTL

**Figure 5:**
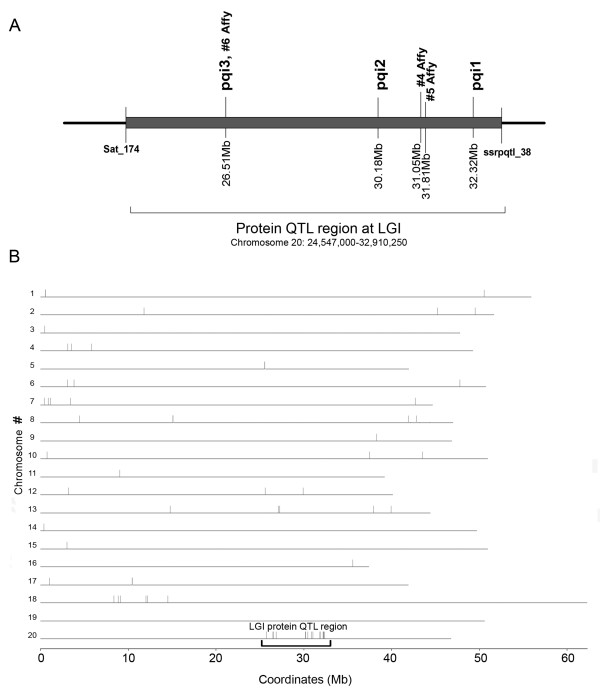
**Location of genes with differentially accumulating transcripts at the LG I protein QTL region in the soybean genome**. (A) Genes with differentially accumulated transcripts identified by Affymetrix^® ^Soy GeneChip at the LG I protein QTL region. (B) The locations of differentially accumulated transcripts found by N-way ANOVA mapped onto the 20 soybean chromosomes. A high-density cluster of transcripts was found at the LG I protein QTL region on chromosome 20.

The three most highly differentially accumulating transcripts identified by Affymetrix^® ^GeneChip were aligned to the soybean genome sequence (version Glyma1, [46]) and found to reside within the borders of the protein QTL region on chromosome 20 (LG I) (Figure 5A). Even though only two of the three were confirmed to accumulate differential levels of transcripts, allelic differences at the segregating QTL region are a potential source for polymorphisms between the two genotypes that could also result in a candidate gene. Three additional differentially accumulating transcripts identified by Affymetrix^® ^GeneChip also mapped to the QTL region, one within 2 kb of pqi2 (Table 3, compare coordinates of #6 and #1-pqi2). Thus, 6 of the 10 differentially accumulating transcripts identified by Affymetrix^® ^GeneChip (Table 3, #1 through #6) resided within the defined boundaries of the protein QTL region at LG I.

Transcripts identified by N-way ANOVA (see Additional file 9) were aligned to the genome sequence to show the range and distribution along the soybean chromosomes (Figure 5B). The soybean genome sequence reveals a general bias toward gene-rich chromosome ends [46], a phenomenon that has been observed in other plant genomes [60]. However, a striking concentration of probes (16 out of 66) mapped to chromosome 20 at the protein QTL region (Figure 5B). The presence of differentially accumulating transcripts in this region is consistent with the development of a near-isogenic line pair that displays variation in seed protein phenotype and segregation of markers within the protein QTL region. Recently, Wei et al. [42] also performed a transcriptome analysis using rice superhybrid *LYP9 *and mapped differentially expressed genes to yield-related QTLs in the rice genome.

### Differentially accumulated transcripts between NILs identified by high-throughput transcriptome sequencing

Because the Soy Genome Affymetrix^® ^GeneChip does not represent the complete set of soybean genes, high-throughput transcriptome sequencing (HTTS) was performed to confirm the microarray data and search for additional candidate genes. Using the same RNA samples prepared for microarray analysis as templates for high-throughput deep sequencing, more than 76 million reads were sequenced, each 36 or 46 nucleotides in length, using the Illumina^® ^Genome Analyzer. Sequences were generated from random priming sites within transcript cDNA from each of the four stages in LoPro and in HiPro, producing more than 7 million reads per stage. Of these reads, more than 20 million aligned uniquely to the genome sequence. The soybean genome sequencing consortium predicted 68,013 gene models and 5,977 additional transposon-like gene models [46]. From that initial set of gene models, the consortium identified 46,430 "high-confidence" genes. In the current seed NILs sequencing effort, 40,352 of the 46,430 (86%) of the high-confidence genes show evidence of expression. An additional 6,078 predicted genes not in the high-confidence set show evidence of expression from the seed NILs data.

Twelve differentially accumulated transcripts between LoPro and HiPro were identified within the LG I protein QTL region with at least a two-fold change in expression at a *P *< 0.001 using HTTS (Table 4). Putative genes were annotated and compared with plant EST and GenBank data sets (Table 4). To further validate the HTTS data, quantitative reverse transcriptase-polymerase chain reaction (qRT-PCR) was performed. Specific primers were designed for four genes from Table 4 with no available corresponding microarray data. Examination of three genes, Glyma20 g19680, Glyma20 g21080, and Glyma20 g21540, by qRT-PCR confirmed higher transcript accumulation levels in LoPro versus HiPro (see Additional file 10), although the standard deviation among biological replicates in Glyma20 g19680 was high. Analysis by qRT-PCR also confirmed higher transcript accumulation levels of Glyma20 g22650 in HiPro versus LoPro.

**Table 4:** Differentially accumulated transcripts between LoPro and HiPro identified by Illumina^® ^high-throughput transcriptome sequencing

#	Comparison	Sequence ID	A	B	Ch	Start	End	Strand	BlastP Description	E-value
1	Overall	Glyma20 g18880	51	0	Gm20	26510968	26513359	-	Mov34-1	2 × 10^-21^
	Stage 3	Glyma20 g18880	15	0	Gm20	26510968	26513359	-	"	"
	Stage 4	Glyma20 g18880	18	0	Gm20	26510968	26513359	-	"	"
										
2	Stage 4	Glyma20 g19620	268	106	Gm20	27706435	27707431	+	no alignments with E-value < 10^-10^	na
										
3	Stage 4	Glyma20 g19630	259	95	Gm20	27706477	27706935	-	no alignments with E-value < 10^-10^	na
										
4	Overall	Glyma20 g19680	24	2	Gm20	27899125	27899596	-	Hsp22.5	5 × 10^-72^
	Stage 3	Glyma20 g19680	24	0	Gm20	27899125	27899596	-	"	"
										
5	Overall	Glyma20 g21030	61	12	Gm20	29984895	29986397	+	Putative ammonium transporter AMT1	0
	Stage 1	Glyma20 g21030	36	4	Gm20	29984895	29986397	+	"	"
										
6	Overall	Glyma20 g21040	40	8	Gm20	29984951	29986210	-	no alignments with E-value < 10^-10^	na
	Stage 1	Glyma20 g21040	27	4	Gm20	29984951	29986210	-	"	"
										
7	Overall	Glyma20 g21080	13	0	Gm20	30044891	30045091	+	ATP synthase D chain	4 × 10^-17^
										
8	Overall	Glyma20 g21140	76	0	Gm20	31078277	30182887	-	no alignments with E-value < 10^-10^	na
	Stage 1	Glyma20 g21140	13	0	Gm20	30178277	30182887	-	"	"
	Stage 3	Glyma20 g21140	38	0	Gm20	30178277	30182887	-	"	"
	Stage 4	Glyma20 g21140	15	0	Gm20	30178277	30182887	-	"	"
										
9	Overall	Glyma20 g21540	32	0	Gm20	30873568	30873806	+	Putative uncharacterized protein	2 × 10^-21^
	Stage 3	Glyma20 g21540	13	0	Gm20	30873568	30873806	+	"	"
										
10	Stage 3	Glyma20 g21780	121	36	Gm20	31386550	31389333	+	Ethylene receptor	0
										
11	Stage 3	Glyma20 g22170	142	46	Gm20	32098751	32103750	+	Glutamyl-tRNA synthetase	0
										
12	Stage 1	Glyma20 g22650	9	42	Gm20	32589230	32589715	+	no alignments with E-value < 10^-10^	na

The sequence identifier (ID) is shown for each numbered gene candidate with differentially accumulated transcripts between genotypes at the LG I protein QTL region. Transcript sequencing read counts for LoPro and HiPro are reported along with the sequence location for the closest predicted gene. The BlastP description is reported for each gene at E-value < 10^-10^. na = not applicable.

### Affymetrix^® ^GeneChip vs. Illumina^® ^high-throughput transcriptome sequencing analysis

Close comparison of the transcripts identified by HTTS (Table 4, #1 and #8) showed the presence of the two most highly differentially accumulated transcripts identified by Affymetrix^® ^GeneChip analysis (Table 3, #1-pqi2 and #3-pqi3). Examination of the coordinates of the most highly differentially accumulated transcripts revealed a distance of 3.7 Mb between pqi2 and pqi3 (Figure 5A). However, the positioning of the soybean target sequence from the Affymetrix^® ^GeneChip for these genes did not directly conform to the predicted gene models in the soybean genome (version Glyma1, [46]).

Interestingly, two pairs of transcripts identified from the Illumina^® ^deep sequencing analysis (Table 4, #2 and #3, #5 and #6) appeared in the same region with overlapping chromosome coordinates but on opposite strands. Transcripts with sequence homology to known proteins included an ethylene receptor and a glutamyl-tRNA synthetase that presented differentially accumulated transcripts at only one stage, as well as a putative ammonium transporter (Table 4). Examination of the available Affymetrix^® ^Soy GeneChip target equivalents that overlapped the region, however, did not provide support for the ethylene receptor and ammonium transporter transcript accumulation differences (Tables 4, see Additional file 11). In all, the union of Affymetrix^® ^Soy GeneChip and Illumina^® ^deep sequencing transcriptome data yielded 13 genes with differentially accumulating transcripts that mapped to the protein QTL region at LG I on chromosome 20 (Tables 3 and 4).

### Genome-wide gene expression coverage

From HTTS of the near-isogenic line pair, a large amount of data was obtained. Uniquely mapped read counts for each genotype at each stage are provided for comparison of transcript accumulation levels at each gene within the defined boundaries of the LG I protein QTL region (see Additional file 11). This list excludes genes annotated as transposon-related. Out of 351 genes on chromosome 20 at the LG I protein QTL region, 252 showed evidence of expression during the seed fill stages examined in this study. The 10 genes in the LG I protein QTL region with the most transcript read counts are listed here (Table 5). Additional file 11 lists all 351 genes in order of total read abundance.

**Table 5:** Ten genes at the LG I protein QTL region with high expression evidence.

Sequence ID	Strand	Start	Stop	A1	A2	A3	A4	B1	B2	B3	B4	Total reads	Top informative Uniprot match	E-value	Description
Glyma20 g19510.1	+	27301033	27301656	1301	575	810	535	1335	1012	404	296	6268	na		na
Glyma20 g21190.1	+	30239601	30241733	290	210	339	212	407	175	225	190	2048	Q94LL1	1 × 10^-112^	Putative 40S ribosomal protein
Glyma20 g22430.1	+	32447769	32449767	240	136	201	194	385	227	156	156	1695	Q6L417	1 × 10^-153^	Putative isopenicillin N epimerase
Glyma20 g22680.1	+	32606778	32610170	245	187	274	146	347	133	132	114	1578	Q38JU3	1 × 10^-100^	ADP ribosylation factor 002
Glyma20 g21230.1	-	30345305	30346033	305	118	234	48	509	185	111	52	1562	Q7G823	5 × 10^-39^	Histone H4
Glyma20 g22090.1	+	31989263	31993579	222	147	243	142	304	133	241	112	1544	Q307Y2	1 × 10^-159^	Putative uncharacterized protein
Glyma20 g21970.1	-	31790681	31793219	140	130	165	121	297	160	229	181	1423	Q9LSW5	5 × 10^-39^	Nicotiana lesion-inducing like
Glyma20 g22600.1	+	32546273	32550838	224	121	247	171	213	103	131	127	1337	Q53VM0	0	Ser/Thr protein kinase - *Lotus japonicus*
Glyma20 g17960.1	-	25068695	25074232	205	103	236	106	190	136	158	122	1256	Q9 M8Z5	0	Putative GTPase
Glyma20 g20010.1	+	28403441	28407180	254	103	148	109	266	124	130	92	1226	Q8VXK6	1 × 10^-47^	F6 protein

Listed in this table are the 10 genes with the most number of uniquely mapped reads contributing to transcript counts (total reads). A1 to A4 correspond to LoPro stages one to four, and B1 to B4 correspond to HiPro stages one to four. na = not applicable, no significant alignment

**Figure 6:**
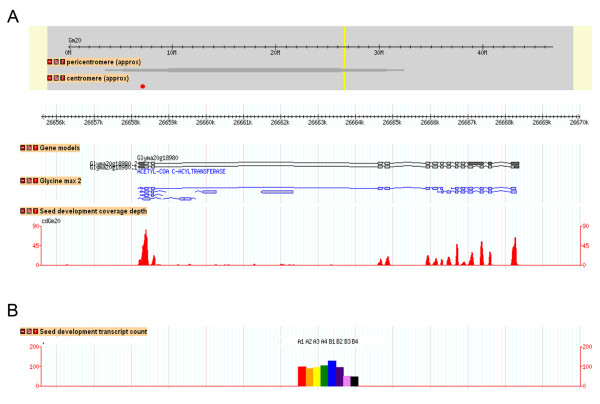
**Soybase Gbrowse HTTS seed development transcript coverage for Glyma20 g18980**. Two different GBrowse annotation tracks displayed at http://soybase.org/gbrowse provide information on coverage depth and location of mapped read counts in relation to the soybean genome sequence. (A) Depicted here is a ~14 kb region from chromosome 20 showing the Glyma20 g18980 gene model "acetyl-CoA c-acyltransferase". Regions with TIGR TA EST data are shown under the "Glycine max 2" track. The "seed development coverage depth" track shows locations and counts of uniquely mapped HTTS reads. The coverage depth track shows the extent of redundancy in coverage at any nucleotide location. (B) The "seed development transcript count" track shows a colored histogram of relative expression counts in each of the eight libraries in this study: A1 to A4 correspond to LoPro stages one to four, and B1 to B4 correspond to HiPro stages one to four. Histograms for each gene are centered under their corresponding gene model.

**Figure 7:**
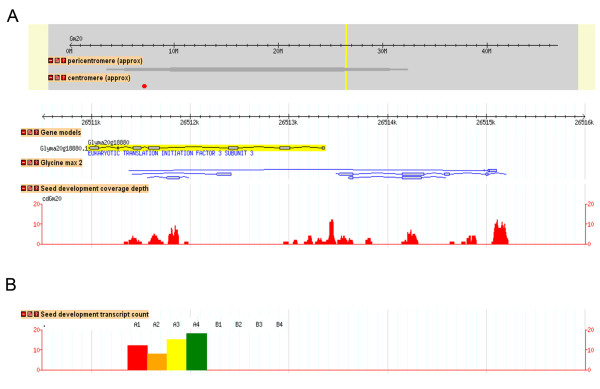
**Soybase Gbrowse HTTS seed development transcript coverage for Glyma20 g18880**. Two different GBrowse annotation tracks displayed at http://soybase.org/gbrowse provide information on coverage depth and location of mapped read counts in relation to the soybean genome sequence. (A) Depicted here is a ~5 kb region from chromosome 20 showing the Glyma20 g18880 gene model annotated here as "eukaryotic translation initiation factor 3 subunit 3". Regions with TIGR TA EST data are shown under the "Glycine max 2" track. The "seed development coverage depth" track shows locations and counts of uniquely mapped HTTS reads. The coverage depth track shows the extent of redundancy in coverage at any nucleotide location. (B) The "seed development transcript count" track shows a colored histogram of relative expression counts in each of the eight libraries in this study: A1 to A4 correspond to LoPro stages one to four, and B1 to B4 correspond to HiPro stages one to four. Histograms for each gene are centered under their corresponding gene model. This gene region (pqi2) shows expression for only four of the eight libraries (values for A1 to A4 only; red, orange, yellow, green).

All HTTS transcript profiles from this study, for all predicted soybean genes, are available at http://soybase.org/gbrowse. Two GBrowse annotation tracks provide information on transcript read coverage and location (Figures 6 and 7). A "seed development coverage depth" track (Figures 6A and 7A) shows locations and counts of uniquely mapped HTTS reads, and a "seed development transcript count" track (Figures 6B and 7B) shows a colored histogram of relative read accumulation counts in each of the eight libraries in this study: A1 to A4 correspond to LoPro stages one to four, and B1 to B4 correspond to HiPro stages one to four. Histograms for each gene are centered under their corresponding gene model. An example of a screenshot depiction of transcript read accumulation coverage is shown for a gene at the LG I protein QTL region, Glyma20 g18980 (Acetyl-CoA C-acyltransferase) (Figure 6). Transcript coverage for Glyma20 g18980 is consistent with the predicted gene model.

Figure 7 shows the gene region for pqi2 where only four of the eight libraries show transcript counts (values for A1 to A4 only; red, orange, yellow, green), consistent with transcript accumulation in LoPro versus HiPro (Tables 3 and 4). The coverage depth track shows the extent of redundancy in coverage at any nucleotide location; for this gene, peak coverage is at approximately 12 reads in any single location. The coverage track shows transcript accumulation at four of the seven predicted exons in the Glyma1.01 gene model for Glyma20 g18880 but also at several other regions outside the predicted gene model. Thus, the HTTS data provide information about expression patterns as well as gene structure and can aid in the improvement of soy gene annotation in the soybean genome while providing genome-wide expression data on seed development.

## Discussion

### Seed protein and oil relationships

It has long been documented that seed protein and oil content are inversely correlated in the soybean seed [28-31,46,61]. Low oil alleles are consistently cotransmitted with high protein alleles in many instances [30,62], and attempts to separate these two traits through chromosomal recombination in the NILs used in this study have not been successful [32]. It has been hypothesized that this relationship may be due to either very tight linkage or pleiotropic effects [27]. Whether one phenotype directly or indirectly results in the other is unknown, and the timing of events regarding differential accumulation of contrasting protein and oil levels in the seed is uncertain. GmDof4 and GmDof11 transcription factors, however, have been reported to activate genes involved in lipid biosynthesis and simultaneously suppress the expression of storage protein genes [56].

Transcription factors have also been shown to influence seed traits in other studies. For example, the putative AP2/EREBP transcription factor WRINKLED1 was found to be involved in the regulation of seed oil accumulation in Arabidopsis [63,64], and a QTL encoding a NAC transcription factor was found to control grain protein and leaf senescence in wheat [15]. In addition, seed mass in Arabidopsis has been shown to be regulated by the APETALA2 (AP2) class of transcription factors [16]. Verdier et al. [65] evaluated the expression of transcription factors throughout seed development of *Medicago truncatula*. They found some 343 transcription factors were expressed equally throughout seed development while 169 had differential expression at one or more stages. Cluster analysis demonstrated six different clusters of transcription factor genes that corresponded to the developmental stages evaluated. Many of the 53 transcription factors that were found to be upregulated in this study during seed development of the soybean NILs were similar to those described by Verdier et al. [65].

Transcriptional suppression of some aspect of seed protein accumulation could be envisioned for the low protein/high oil NIL homozygous for the *G. max *allele of the LG I QTL. However, transcriptional suppression of seed oil accumulation in the NIL homozygous for the *G. soja *allele (assuming a repulsion-based pleiotropy of the two alleles of the candidate gene underlying this QTL) would be envisioned to occur in a time frame late in seed fill. This assumption is due to the observation that the rate of seed oil accumulation in HiPro did not differ from that of LoPro until the last stage of seed fill (Figure 2). Although HiPro matures slightly earlier and generally yields less seed than LoPro [27,32], these differences do not fully account for the striking differences in NIL seed protein content observed at the early stages of seed fill. Whether additional differences in the morphology or composition of the seed exist between the near-isogenic lines remains to be seen. Further detailed investigation is in progress to study the temporal and spatial distribution and partitioning of candidate gene expression that may govern the relationship between protein and oil accumulation in the developing soybean seed.

### Processes and pathways influencing seed content

Comprehensive evaluation of seed transcripts through microarray analyses have been reported for Arabidopsis [34], *Medicago truncatula *[4,38], barley [5,41], and wheat [40]. These studies, in common, report differential expression of hundreds of genes at one or more stages of seed development involved in processes related to carbon and nitrogen metabolism, protein processing, transport of nutrients, organ development (transcription factors), signal transduction, and phytohormone balance. The transcript accumulation patterns we observed during NILs seed fill by GeneChip^® ^microarray data were consistent with these studies.

Prior studies have demonstrated the transcription and accumulation of both mRNA and protein for beta-conglycinin and glycinin genes during the seed fill stage of seed development [66-68]. Transcripts for these seed storage proteins were identified during seed fill with particular abundance in the HiPro line (Tables 1 and 2, see Additional files 2, 3, 4, 5, 6, 7). Additional classes of genes with roles in seed development and maturation, flavonoid metabolism, and sucrose binding were also identified. Proteome analyses of the seed filling stages in soybean have provided support for the presence of gene transcripts found in this study with the detection of proteins associated with protein destination and storage, metabolism, and disease/defense [3]. Expression of different protein isoforms have been shown to display different accumulation trends, and the activities of many genes may have multiple roles during seed filling. This phenomenon may be reflected in the increase and decrease in accumulated transcripts of lipoxygenase-related genes in this study, consistent with proteomic data on various lipoxygenases in the developing soybean seed [3].

Carbon metabolism directed toward oil and protein deposition plays an important role in seed quality. Changes in seed protein or oil in many plant species have been linked to the activity of acetyl-CA carboxylase (ACCase) [69-71] and phosphoenolpyruvate carboxylase (PEPC) [72-75]. Recent proteomic and microarray studies have shown the presence of peptides and transcripts for both enzymes during seed development [6,38,76]. Overexpression of Arabidopsis acetyl-CoA carboxylase led to increased oil content of *Brassica napus *seeds [70] and potato tubers [77]. The acetyl-CoA carboxylase gene has also been associated with a major groat oil content QTL [78]. In addition, inhibition of plastid acetyl-coA carboxylase resulted in lower seed oil [79]. In soybean, a significant correlation was found between phosphoenolpyruvate carboxylase activity and seed protein and oil concentrations [75], although this correlation was found to be higher for seed protein. Furthermore, overexpression of phosphoenolypruvate carboxylase in *Vicia narbonensis *seed was shown to increase seed storage capacity and protein content [80]. Although we found no significant differences in transcript expression of ACCase and PEPC between NILs, we observed that transcripts corresponding to several forms of ACCase and PEPC were expressed at all stages of seed development in this study (data not shown). Interestingly, some forms of ACCase were expressed at higher levels in the seed than others. Such data may reflect the importance of enhanced isoforms of ACCase and PEPC in seed development compared to isoforms expressed elsewhere in the plant.

Impaired storage metabolism has been linked with decreased sucrose levels [2], and sucrose may affect carbon flux at the transcriptional or post-transcriptional levels [81]. Studies have shown the importance of photosynthesis in seed filling metabolism [82] and for the biosynthesis of seed storage products [83,84] consistent with the wide array of photosynthesis-related genes detected during seed fill in this study. Regulation of protein destination, storage, and proteolysis, as well as metabolic and photosynthetic pathways, may contribute to the contrasting seed phenotypes seen in the NIL pair.

Additional transcript accumulation changes have been documented during seed development. A heat shock protein and peptide transporter were among the annotations of the transcripts with the greatest fold change increases from stage one to stage four in LoPro (see Additional file 3). Both a peptide transporter and heat shock-related proteins were previously found to increase dramatically during seed development in a high oil soybean line [85]. Down-regulation of lipoxygenases and sucrose UDP-glycosyltransferase during seed development in a high oil soybean line of a previous study [85] is also consistent with the detection of down-regulated lipoxygenase and UDP-glycosyltransferase transcripts in LoPro (see Additional file 3). The transcription accumulation patterns of these genes may be a feature common to soybean lines with high oil phenotypes.

### Candidates for regulation of seed protein and oil

We identified 14 genes mapping to the protein QTL region at LG I that may play a role in the regulation of seed protein and oil. Thirteen of these 14 genes displayed differentially accumulating transcripts. Of these 13, 11 were found at high levels in the low protein line with low or no detectable levels in the high protein line. Based on sequence homology searches to protein databases, these candidates include a potential regulatory protein in the Mov34-1 family, a heat shock protein Hsp22.5, and an ATP synthase (Table 3).

Although the Mov34-1 candidate appeared to possess versatile domains for the potential regulation of multiple processes, transcripts isolated from this candidate region contained numerous stop codons, raising the possibility of non-coding genes. The same was true for a number of the other candidates and may account for the high percentage of genes with no significant E-value returns to the Uniprot protein database [86]. There is increasing evidence for the role of riboregulators, either as long non-protein coding RNAs or processed into small RNAs in plant development [87], and these molecules may play a role in seed protein and oil accumulation. Two pairs of genes among the candidates (Table 4) were found to possess overlapping transcripts; one possibility is that these overlapping transcripts form double-stranded RNAs that may be processed into small RNAs [88].

Evidence for the expression of heat shock proteins during the stress-independent development of the seed has previously been observed [89,90]. Interestingly, heat shock protein genes were found to be expressed at higher levels in the low protein line of a near-isogenic line pair in barley [5], a phenomenon also observed in the LoPro line of this study. Previous studies have detailed an indirect relationship among the accumulation of storage proteins, lipid biosynthesis, and photosynthesis in the seed, correlating to the availability and distribution of ATP [83,84,91,92]. Further investigation into the modulation of ATP synthase levels on energy status and storage product accumulation in the soybean seed will shed light on the potential role for ATP synthase as a candidate gene. Currently, the occurrence of additional candidate genes from even earlier stages of seed development is being evaluated through differential analysis of transcriptome profiles of the near-isogenic line pair.

### Potential modes of regulation for seed protein and oil

The LoPro line was converted into the HiPro line upon inheritance of a *G. soja *allele at the LG I protein QTL region. However, the LoPro line is also the high oil line, and a number of scenarios may explain how gene expression differences relate to variation in protein and oil phenotypes in the seed.

Protein content may be positively regulated by the expression of a gene that increases protein production in HiPro. Alternatively, protein content may be negatively regulated by expression of a gene in LoPro that inhibits or reduces protein accumulation and thus allows for increased oil accumulation. Significant protein differences would then be observed at an earlier stage than oil differences, as in Figure 2. Inhibition of protein accumulation could take place at many levels, including transcriptional and post-transcriptional control and regulation of protein synthesis, transport, and turnover. The presence of candidate genes with non-coding segments raises the possibility of regulation at the transcriptional level that may affect the transcription of genes outside the list of candidates shown in this study.

Differences in transcriptome profiles may correlate directly or indirectly with the differences in protein and oil accumulation between the NILs. Previous studies have shown that seed storage proteins are largely controlled by transcriptional regulation during the seed fill stage (reviewed in [39]). Extensive analysis of cis-regulatory elements of seed storage proteins has demonstrated interaction of these elements with bZIP and MYB factors [39,93-96]. Transcription of a candidate gene in LoPro may result in negative regulation of transcriptional regulators or key factors involved in high protein accumulation. The presence of sequence polymorphisms in gene sequences or promoter regions within the segregating region of the protein QTL may account for the low or absent levels of differentially accumulating gene transcripts in HiPro versus LoPro (Table 4).

In an alternative scenario, oil content may be regulated. Gene expression or transcript accumulation leading to a higher oil phenotype may act in concert with other factors to directly or indirectly lead to reduced protein accumulation. Genes regulated by transcription factors could initiate this effect. In support of this model, batch analysis of the promoter regions of the genes with the greatest differentially accumulated transcripts between the NILs revealed a number of transcription factor binding sites and seed-specific motifs (data not shown). A regulatory factor expressed in the high oil LoPro line may activate higher oil synthesis or accumulation pathways. This is consistent with the greater abundance of candidate gene transcript accumulation seen in LoPro (Table 4). Inheritance of a *G. soja *allele that does not allow for expression or accumulation of the high oil gene could account for the low oil and high protein phenotype in HiPro.

### Utility of the HTTS dataset for understanding the soybean genome

Although we focused on the transcripts derived from the LG I region of the genome in this study, the high-throughput transcriptome sequencing data set we obtained compiles greater than 76 million reads and 2.76 × 10^9 ^nucleotides of transcript data and is an excellent resource for increasing our understanding of the soybean genome. The use of HTTS in conjunction with microarrays allowed us to detect a more comprehensive set of soybean gene transcripts. Our observation that 86% of gene transcripts in soybean were present during seed development greatly extends previous microarray-dependent seed development studies.

Recent reports demonstrate the value of high-throughput transcriptome sequencing in eukaryotes for identification of novel transcripts and transcript isoforms, untranslated regions, and gene structures, leading to improved genome annotation [97-100]. For the soybean genome, current gene models using the 8× genome sequence assembly (version Glyma1, [46]) were predicted based on protein coding sequences. By comparison, our transcriptome dataset encompasses both protein coding and non-protein coding sequences and will be useful for identification of transcripts outside of gene models. Analyses of our dataset also show evidence for the existence of novel transcript isoforms, including alternative splicing, between genotypes and among seed stages (data not shown). Moreover, beyond the detection of feature polymorphisms reported here, a comparative analysis of common transcripts between soybean lines will provide a multitude of single nucleotide polymorphisms useful in following agronomic traits in breeding populations. Currently we are analyzing high-throughput sequencing of transcripts from many soybean tissues. That data, along with the seed transcriptome data, will compile an atlas of gene expression for soybean.

## Conclusions

This study provided the rare opportunity to intersect structural mapping and molecular profiling studies. Here, we compared the transcript abundance profiles of the developing seed from a soybean NIL pair with contrasting seed protein and identified gene candidates at the LG I protein QTL for potential involvement in the regulation of protein content in the soybean seed. The entire transcriptome sequencing dataset generated from this study is also provided as a valuable resource.

Control of protein and oil accumulation in the seed occurs at many different levels and is likely influenced by more than one gene. Of the candidates genes identified in this study, any combination could be responsible for the observed change in protein and oil and phenotypes conditioned by the alleles of the LG I QTL. Other protein/oil QTLs have been identified in QTL mapping studies, but the LG I QTL is of great interest because its additive effect on seed protein and oil is the largest of any QTL identified to date. The models presented here are compatible with the role of additional genes and pathways as well as mixed models for control of seed protein and oil. Resources that include the availability of additional recombinants and the use of markers derived from this study will allow for further demarcation of the QTL region. Further studies are being conducted on additional mapping populations to dissect the relationship between protein and oil levels, and functional studies are under way to identify and validate the role(s) of candidate genes in the accumulation of protein and oil in the seed.

## Methods

### Physical mapping of the QTL region

The QTL flanking SSRs from a previous genetic study [27], Satt239 and Satt496, as well as three other SSR markers (Sat_174, Sat_219, and Satt700) in the vicinity of the putative QTL region were used to PCR (polymerase chain reaction) screen multi-dimensional pools of the soybean [*Glycine max *(L.) Merrill] 'Williams 82' and 'Fairbault' BAC libraries. BAC clones were end-sequenced using M13 forward and reverse primers at the Iowa State University DNA sequencing and synthesis facility. The BAC libraries were then rescreened by PCR using primers designed from BAC end-sequences, and the BAC contigs were extended by chromosome walking. BACs were fingerprinted using restriction enzymes EcoRI and AccI, and BAC overlap was confirmed by FPC (FingerPrinted Contig) 4.6.4 [101]. BAC overlap was also verified by PCR using primers from BAC end-sequences. A minimal tiling path of BACs were identified and subsequently sequenced.

### BAC sequencing and assembly

BAC DNA was isolated by plasmid midi-prep (Qiagen, Valencia, CA). Random sheared BAC DNA was size selected for 2 to 3 kb and subcloned onto vector pCR^® ^4Blunt-TOPO^® ^using the TOPO^® ^shotgun subcloning kit (Invitrogen). The recombinant plasmids were transformed into competent TOP10 *E. coli *cells by electroporation. Transformants were isolated on LB plates containing kanamycin. Subclones were sequenced using M13 forward and reverse primers at the Iowa State University DNA sequencing and synthesis facility. Vector trimming, removal of poor quality reads, and sequence assembly were carried out using the program SeqManII (DNASTAR, Inc.) using default parameters with a minimum match percentage of 95% for sequence assembly. Contigs were ordered based on the positions of the reverse and forward reads of the same subclones. Sequence gaps were filled either by complete sequencing of the subclones that spanned the gaps or by PCR amplification across the gap using BAC DNA followed by complete sequencing of the PCR products.

### Demarcation of the QTL region

The BAC sequences were aligned to the sequence scaffolds (version Glyma0 and Glyma1, [46]) of the genome sequence http://www.soybase.org by BLASTN [102]. All the BAC sequences showed the best match to chromosome 20. Additional SSRs were identified from within the putative QTL region and tested for polymorphism between lines A81-356022 and PI468916. All the polymorphic SSRs were initially amplified from 'Williams 82' (the reference genotype for which the genome sequence is available) to verify that the primers were amplifying products of expected sizes and therefore were targeted to the QTL region. Further, the polymorphic markers from within the QTL region were screened for segregation in the population P-C609-45-2 described below that segregates for only the 3 cM region surrounding the QTL [32]. This SSR analysis identified the recombination break points for a more precise positioning of the QTL region.

### Development of NILs

NILs were developed by introgression of the high protein QTL allele on LG I from *G. soja *PI468916 into *G. max *A81-356022 for BC_5_F_5 _populations [25,32]. The NIL population P-C602-15-6 contained 53 lines. A single BC_5_F_5 _plant from P-C609-45-2 that was heterozygous for the Satt496 marker in the LG I protein QTL region was designated as P-C609-45-2-2 and produced 39 BC_5_F_6 _lines [32]. A NIL pair (LD04-15154 = HiPro and LD04-15146 = LoPro) derived from P-C609-45-2-2 was chosen from among the BC_5_F_6_lines for segregation at the LG I protein QTL region for marker Satt496 and for corresponding high and low seed protein phenotypes from field trials. Additional markers for segregating and non-segregating regions were confirmed for the NIL pair and verified in the parental lines as described above.

### Plant growth and experimental design

In order to minimize uncontrolled environmental conditions, the NIL pair consisting of LoPro and HiPro was grown in growth chambers at the University of Minnesota. Soybeans were initially grown in the growth chamber at a photoperiod of 14/10 and thermocycle of 22°C/10°C. Day length and temperature were monitored to mimic Illinois field growing conditions. Contrasting NILs were planted in staggered pairs, and three biological replicates were conducted following a complete random design. Each replicate was harvested at the same time of day and consisted of seed samples at four developmental stages pooled from three plants. Samples were harvested from the NILs in parallel and flash frozen in liquid nitrogen before storage at -80°C. Stage one corresponded to 25 to 50 mg, stage two to greater than 50 to 100 mg, stage three to greater than 100 to 200 mg, and stage four to greater than 200 to 300 mg seed.

### Seed protein and oil analysis

The NILs were grown to maturity, and seed from both genotypes was harvested at each of the four stages. Seed was also harvested from the final mature seed stage, and replicate samples were pooled by stage and genotype and analyzed for protein and oil at the Agricultural Experiment Station chemical laboratories at the University of Missouri-Columbia (UMC). Soybean seed was weighed before and after freeze-drying and then submitted to UMC for laboratory analysis. A combustion protocol using AOAC Official Method 990.03 [103] was used to analyze protein concentration in the soybean seed samples. Oil levels were determined by ether extraction following AOAC Official Method 902.39A [103].

### RNA isolation

Seed was ground with liquid nitrogen by mortar and pestle. Total RNA was isolated by a modified TRIzol^® ^(Invitrogen) protocol [104] and then digested with on-column RNase-free DNase (Qiagen) and purified by RNeasy column (Qiagen). RNA quality was evaluated by gel electrophoresis, spectrophotometer, and Agilent 2100 bioanalyzer.

### Microarray preparation and processing

Processing and labeling of RNA samples was performed by Qiagen^® ^Target Prep Robot at the Biomedical Image Processing Facility at the University of Minnesota. Synthesis of cDNA was performed using the SuperScript Double-Stranded cDNA Synthesis Kit (Invitrogen) on 5 μg of total RNA from each sample, and biotinylated cRNA was produced using the Enzo BioArray HighYield RNA transcript labeling kit (Enzo Life Sciences, Farmingdale, NY, U.S.A.) in the presence of biotinylated UTP and CTP. Samples were purified by RNeasy kit (Qiagen), quantified by Biotek^® ^Synergy HT plate reader, and chemically fragmented using the Affymetrix^® ^GeneChip sample cleanup module. Samples were then hybridized to the Soy Genome Affymetrix^® ^GeneChip using an Affymetrix^® ^Hybridization Oven 640, and arrays were washed on an Affymetrix^® ^Fluidics Station 450 using Affymetrix^® ^fluidics protocol EukGE-WS2v4_450. Details of this protocol can be found in the Affymetrix^® ^Genechip Expression Analysis Technical Manual, Section 2, Chapter 3 http://www.affymetrix.com/support/downloads/manuals/expression_analysis_technical_manual.pdf.

### Microarray data processing and analysis

The Soy Genome Affymetrix^® ^GeneChip http://www.Affymetrix.com containing greater than 37,500 probesets and representing 35,611 soybean transcripts [105], was used to assess gene expression. Microarray data were analyzed using Expressionist Pro software from Genedata Inc. Raw data in the form of .CEL files from the Affymetrix^® ^GeneChip were uploaded to the platform, and the robust microarray analysis (RMA) algorithm [106] was used to condense and normalize all soybean probeset data with a median of ten thousand. Correlation coefficients for the three biological replicates assessed per sample genotype and time point (stage) ranged from 0.9809 to 0.9982 after normalization. The detection quality was set to a value of one to ensure that all probe sets were considered. MAS5.0 [107] data condensation and normalization were also performed for comparison purposes. An FDR value was computed for each *P *value [48]. Differentially accumulated gene transcript lists were produced at false discovery rates estimated at 5% or less. Microarray data sets were deposited under experiment GM11 in the Plant Expression database (PLEXdb) [108].

### SFP identification

Single Feature Polymorphisms (SFPs) were identified using a method [57] based on the Li-Wong model [58]. This method compares the relative probe intensities of each of the 11 probes on the Affymetrix^® ^GeneChip between genotypes. Statistical analysis of the probe affinity difference was calculated using the feature intensity of the perfect match (PM) probes. Given the raw intensity (S) of each feature (probe) determined by the gene expression level (I), the affinity (A) between the target transcript and the probe, and random error (E) [58,109-111], the equation can be modeled as A_tij _+ E_tij _= S_tij _- I_ti _. Here, S_tij _is the raw PM intensity and I_ti _is derived from the RMA expression value of each gene for the designated genotype (t), probe set (i), and probe (j), where Et_1_ij ≈ Et_2_ij, since E is an independent identically distributed error with a mean of zero. The Bioconductor Affymetrix^® ^package was used to extract PM intensity and to calculate RMA expression, and the Bioconductor Siggenes package was used to evaluate all probe sets.

### Gene annotation

Genes were annotated using the Affymetrix^® ^GeneChip Soybean Genome Array Annotation http://www.soybase.org/AffyChip from SoyBase and The Soybean Breeder's Toolbox in conjunction with annotations from the HarvEST soy assembly website http://www.harvest-web.org. Unannotated genes were individually scanned by BLASTX and TBLASTX at an E-value cutoff of 10^-4^. The UniProt protein database [86], the Pfam protein database [112], the *Arabidopsis thaliana *genome database (TAIR, http://www.arabidopsis.org), and the *Medicago truncatula *genome database http://www.medicago.org were used for annotation purposes. TAIR gene ontology (GO) and GO slim annotations [113] were provided for each Arabidopsis match. BLASTP results with an E-value of less than 10^-10 ^were used to describe gene sequences referenced on the soybean genome (version Glyma1, [46]).

### Statistical analysis of gene ontology and expression

The consensus sequences of the soybean genes on the Soy Genome Affymetrix^® ^GeneChip were compared to the most recent release of predicted genes in the Arabidopsis genome (TAIR v. 8, http://www.arabidopsis.org) using TBLASTX (E < 10^-4^, [102]). The top Arabidopsis gene was used to query the Arabidopsis gene ontology (TAIR ATH_GO_GOSlim.20080308, http://www.arabidopsis.org) [113]. A database was created linking each Affymetrix^® ^probe to the most similar Arabidopsis gene (E < 10^-6^) and its corresponding gene ontology information [114]. Custom Perl scripts were used to mine the database for the GO slim annotations of the differentially expressed genes of interest.

To determine if particular GO slim categories were over-represented in our expression data, the number of genes matching each GO slim category was determined. This procedure was repeated to determine the number of genes matching each GO slim category for all the soybean consensus sequences represented on the chip. For each GO slim category, Fisher's exact test [115] was used to compare the number of expressed genes in the GO slim category, the number of genes not differentially expressed in the GO slim category, the number of differentially expressed genes outside the GO slim category, and the number of genes not differentially expressed and outside the GO slim category. To correct for oversampling, a Bonferroni correction [116] was used to adjust the two-tail probability *P *value. The *P *value obtained using Fisher's exact test was multiplied by the total number of GO categories represented on the Affymetrix^® ^Soy GeneChip. Only *P *values more significant than 0.05 after Bonferroni correction are reported. Further, only GO Slim categories that were significantly over-represented in the expression data are reported.

### qRT-PCR analysis

Quantitative RT-PCR was performed and analyzed using the Applied Biosystem Real-Time PCR system. Gene-specific primers spanning a maximum of 150 bp were designed using Primer Express^® ^software (Applied Biosystems). Gene-specific actin primers were also used for control and calculation purposes. Template cDNA was synthesized from total RNA using a reverse transcription cDNA synthesis kit (Invitrogen). Reactions with no reverse transcriptase were performed as controls. Quantitative RT-PCR was performed in three replicates in a 96-well plate using SYBR^® ^Green (BioRad) at 35 cycles. Results were calculated using the comparative C_T _method to evaluate gene expression in LoPro vs. HiPro or HiPro vs. LoPro with respect to the actin control at each stage.

### Transcriptome sequencing

Total RNA from stages one through four of LoPro and HiPro was used for Illumina^® ^sequencing. Poly A+ RNA was isolated from total RNA through two rounds of oligo-dT selection (Invitrogen Inc., Santa Clara, CA). The mRNA was annealed to high concentrations of random hexamers and reverse transcribed. Following second strand synthesis, end repair, and A-tailing, adapters complementary to sequencing primers were ligated to cDNA fragments. Resultant cDNA libraries were size fractionated on agarose gels, and 250 bp fragments were excised and amplified by 15 cycles of polymerase chain reaction. Ensuing libraries were quality assessed using the Agilent 2100 bioanalyzer platform and sequenced for 36 or 46 cycles on an Illumina^® ^Genome Analyzer DNA sequencing instrument using standard Illumina^® ^procedures.

### Sequencing data processing and analysis

To process the data for analysis, files were mirrored to an off-instrument computer using the Illumina^® ^platform to perform image analysis, base-calling, and per base confidence scores. Individual transcript tags were identified, counted, and scored for uniqueness. Sequence reads were then aligned against the 8X soybean genome sequence assembly (version Glyma1, [46]) using MAQ [117]. Read mappings were retained if they met the following criteria: they had a mapping quality of 99, or had no mismatches, or the sum of the quality scores of the mismatched bases was less than or equal to six (using Phred quality scores). If a read mapped equally well to multiple locations (therefore producing a mapping score of zero), MAQ randomly returned one of the locations. Counts were made with respect to predicted genes in the Glyma1.01 annotation by incrementing the count for a gene when any part of a read overlapped the longest splice variant of the gene model. Counts per gene and tissue are displayed in an "expression" GBrowse track at http://soybase.org/gbrowse, and all reads, without regard to gene boundaries, are displayed in another expression GBrowse track. The significance of gene expression between treatment pairs (e.g., A1 LoPro vs. A1 HiPro) was tested for each gene by comparing the normalized values for that gene against a two-tailed binomial distribution using a P-value of 0.001. Normalizations were calculated by multiplying the count values in each treatment by the experiment-wide average over the treatment sum. The test for significance for a given gene, with counts C1 and C2 (and C1 < C2), is whether the probability of observing C1 or fewer counts out of C1 + C2 trials (counts observed for genes from both treatments) is less than or equal to 0.0005 (for a two-tailed threshold of 0.001).

### Soybean physical mapping

Sequence information was downloaded from the latest soybean genome sequence assembly (version Glyma1, [46]) to obtain 50,527 unique soybean gene identifiers with chromosome locations. Soy Genome Affymetrix^® ^GeneChip probeset consensus sequences were retrieved http://www.Affymetrix.com for a total of 61,035 cDNA sequences. The NCBI blast program [102] was used to align Affymetrix^® ^Soy GeneChip probeset consensus sequences against the soybean cDNA database (Glyma1.cDNA.fa, http://www.phytozome.net/soybean.php) containing 75,778 sequences. With the blastn search tool, the match matrix BLOSUM62 was used with the following parameters: mismatch penalty -3, E-value 10^-5^, and bit score 100. This analysis aligned 36,406 Affymetrix^® ^soy identifiers to soy genome identifiers with chromosome locations. The genome sequences and probesets with chromosome information were imported into the genome browser of GeneSpring version 7.3.1 http://www.Agilent.com for mapping of genes and probeset locations onto chromosomes.

## Authors' contributions

YTB participated in experimental concept and design and performed GeneChip experiments, data analysis, and interpretation. BJ was responsible for delineating the LG I protein QTL genome boundaries. YTB and BJ were equal main authors responsible for writing the manuscript. SBC also contributed to writing of the manuscript. SBC, GDM, ADF, and NW were responsible for high-throughput sequencing, mapping transcripts to the genome, and sequencing data analysis and interpretation. MAG participated in data analysis and developing gene ontogeny data. BWD, GJM, JES, and RCS participated in experimental design and concept. ZJT contributed to genomic data analyses. WWX contributed to microarray data analysis and performed single feature polymorphism analysis on microarray data. CPV participated in experimental concept and design, data analysis and interpretation, and writing of the manuscript. All authors contributed to editing of the manuscript. All authors read and approved the final manuscript.

## Supplementary Material

Additional file 1 **Details of the SSR markers derived from the BAC sequences and the whole genome sequence spanning the LG I QTL region**. Forward and reverse primer sequences and start and end sites for SSR markers are listed with PCR product size in 'Williams82' and segregation status in NILs. Click here for file

Additional file 2 **Differentially accumulating transcripts between stage three and stage one within genotypes**. Affymetrix^® ^Soy Genechip probesets with differential expression values between stage three and stage one are listed with the ratio of mean values from three biological replicates at stage three versus stage one within LoPro (tab A3vA1) or HiPro (tab B3vB1) along with Uniprot, Arabidopsis, and *M. truncatula *alignment descriptions. Click here for file

Additional file 3 **Differentially accumulating transcripts between stage four and stage one within genotypes**. Affymetrix^® ^Soy Genechip probesets with differential expression values between stage four and stage one are listed with the ratio of mean values from three biological replicates at stage four versus stage one within LoPro (tab A4vA1) or HiPro (tab B4vB1) along with Uniprot, Arabidopsis, and *M. truncatula *alignment descriptions. Click here for file

Additional file 4 **Transcripts upregulated from stage one to stage four in both genotypes**. Affymetrix^® ^Soy Genechip probesets with higher expression values in stage four than stage one in both HiPro and LoPro are listed with mean values from three biological replicates at each stage and Uniprot, Arabidopsis, and *M. truncatula *alignment descriptions. Click here for file

Additional file 5 **Transcripts downregulated from stage one to stage four in both genotypes**. Affymetrix^® ^Soy Genechip probesets with lower expression values in stage four than stage one in both HiPro and LoPro are listed with mean values from three biological replicates at each stage and Uniprot, Arabidopsis, and *M. truncatula *alignment descriptions. Click here for file

Additional file 6 **Transcripts with greater than four-fold change in accumulation differences between stage four and stage one in LoPro**. Affymetrix^® ^Soy Genechip probesets with greater than four-fold change in accumulation differences between stage four and stage one in the LoPro genotype are listed with mean values at stage one and stage four, ratio of mean values, and Uniprot, Arabidopsis, and *M. truncatula *alignment descriptions. Click here for file

Additional file 7 **Transcripts with greater than four-fold change in accumulation differences between stage four and stage one in HiPro**. Affymetrix^® ^Soy Genechip probesets with greater than four-fold change in accumulation differences between stage four and stage one in the HiPro genotype are listed with mean values at stage one and stage four, ratio of mean values, and Uniprot, Arabidopsis, and *M. truncatula *alignment descriptions. Click here for file

Additional file 8 **Overrepresented gene categories with transcript accumulation changes during seed fill**. Gene ontology (GO) categories that are overrepresented in the NIL genotypes LoPro and HiPro are shown next to the stages compared and the direction of trend changes. For *Stage comparison: 1 to 4 *and *Trend: decrease*, the trend of transcript accumulation decreases from stage one to stage four. The GO term identifier is indicated along with the functional categorization. BP = biological process. MF = molecular function. # of genes = number of genes represented by the Affymetrix^® ^Soy GeneChip with transcript accumulation changes. Click here for file

Additional file 9 **Differentially accumulating transcripts between NILs identified by N-way ANOVA analysis of Affymetrix^® ^Soy GeneChip microarray data**. Affymetrix^® ^Soy Genechip probesets with differential expression values between NILs detected by N-way ANOVA analysis are listed with Uniprot alignment descriptions. Click here for file

Additional file 10 **Quantitative RT-PCR for four genes detected as differentially accumulated in the genomic segment containing the LG I protein QTL by Illumina HTTS**. Gene identifiers refer to genes with differentially accumulated transcripts listed in Table 4. (A) Glyma20 g19680, Glyma20 g21080, and Glyma20 g21540 transcripts were detected at higher levels in LoPro than HiPro (Table 4). Transcript level fold changes for Glyma20 g19680, Glyma20 g21080, and Glyma20 g21540 were compared between LoPro and HiPro lines with reference to an actin control in stage 3 seed by qRT-PCR. (B) Glyma20 g22650 transcripts were detected at higher levels in HiPro than LoPro in stage 1 seed (Table 4). Transcript level fold changes for Glyma20 g22650 were compared between HiPro and LoPro lines with reference to an actin control in stage 1 seed by qRT-PCR. Click here for file

Additional file 11 **HTTS read counts for genes within the LG I protein QTL region**. Genes within the LG I protein QTL region on chromosome 20 are listed in order of those with the greatest to least number of total read count evidence from Illumina HTTS of seed tissue at four seed stages in both genotypes. Read counts are normalized, and genes with transposon-related annotations have been removed from this list. Click here for file
