# Supplementary material for: Complementary genetic and genomic approaches help characterize the linkage group I seed protein QTL in soybean
Source: BMC Plant Biol. 2010 Mar 3;10:41. doi: 10.1186/1471-2229-10-41 (PMC2848761; doi:10.1186/1471-2229-10-41)

**Additional file 10: Quantitative RT-PCR for four genes detected as differentially accumulated in the genomic segment containing the LG I protein QTL by Illumina HTTS.** Gene identifiers refer to genes with differentially accumulated transcripts listed in Table 4. (A) Glyma20g19680, Glyma20g21080, and Glyma20g21540 transcripts were detected at higher levels in LoPro than HiPro (Table 4). Transcript level fold changes for Glyma20g19680, Glyma20g21080, and Glyma20g21540 were compared between LoPro and HiPro lines with reference to an actin control in stage 3 seed by qRT-PCR. (B) Glyma20g22650 transcripts were detected at higher levels in HiPro than LoPro in stage 1 seed (Table 4). Transcript level fold changes for Glyma20g22650 were compared between HiPro and LoPro lines with reference to an actin control in stage 1 seed by qRT-PCR.

**A**

**
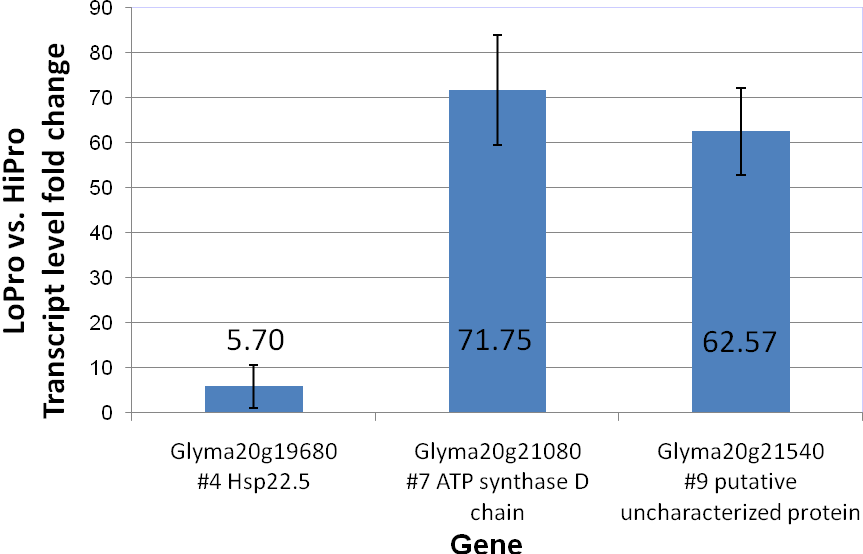
**

**B**


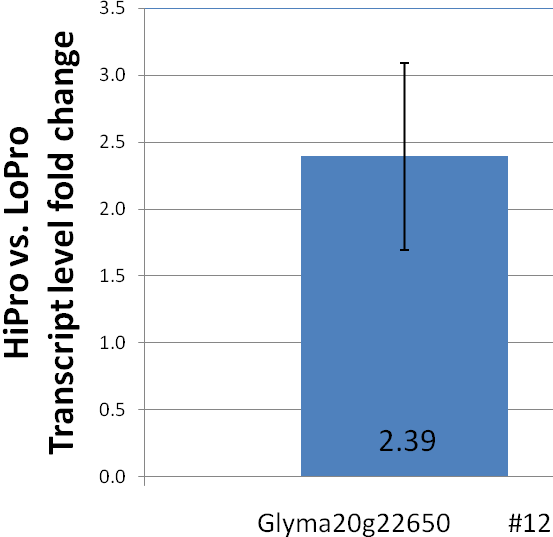

Supplement: Additional file 10 — Quantitative RT-PCR for four genes detected as differentially accumulated in the genomic segment containing the LG I protein QTL by Illumina HTTS. Gene identifiers refer to genes with differentially accumulated transcripts listed in Table 4. (A) Glyma20 g19680, Glyma20 g21080, and Glyma20 g21540 transcripts were detected at higher levels in LoPro than HiPro (Table 4). Transcript level fold changes for Glyma20 g19680, Glyma20 g21080, and Glyma20 g21540 were compared between LoPro and HiPro lines with reference to an actin control in stage 3 seed by qRT-PCR. (B) Glyma20 g22650 transcripts were detected at higher levels in HiPro than LoPro in stage 1 seed (Table 4). Transcript level fold changes for Glyma20 g22650 were compared between HiPro and LoPro lines with reference to an actin control in stage 1 seed by qRT-PCR. [file 1471-2229-10-41-S10.DOC]
